# Comparative pharmacological characterization of D_1_-like dopamine receptors from *Anopheles gambiae*, *Aedes aegypti* and *Culex quinquefasciatus* suggests pleiotropic signaling in mosquito vector lineages

**DOI:** 10.1186/s13071-016-1477-6

**Published:** 2016-04-06

**Authors:** Catherine A. Hill, Trevor Doyle, Andrew B. Nuss, Karin F. K. Ejendal, Jason M. Meyer, Val J. Watts

**Affiliations:** Department of Entomology, Purdue University, 901 W. State St., West Lafayette, IN 47907-2089 USA; Department of Medicinal Chemistry and Molecular Pharmacology, Purdue University, 575 Stadium Mall Drive, West Lafayette, IN 47907-2091 USA; Present address: Department of Agriculture, Nutrition, and Veterinary Science, University of Nevada, Reno, NV 89557 USA; Present address: Department of Biotechnology, Monsanto Company, Chesterfield, MO 63017 USA

**Keywords:** *Anopheles gambiae*, Malaria mosquito, G protein-coupled receptor, Dopamine, Antagonist, Signaling, Novel insecticide

## Abstract

**Background:**

Small molecule antagonists of mosquito dopamine receptors (DARs) are under investigation as a new class of vector-selective insecticides. Antagonists that inhibit the D_1_-like DARs *Aa*DOP2 and *Cq*DOP2 from the mosquitoes *Aedes aegypti* L. and *Culex quinquefasciatus* Say, respectively*,* also cause larval mortality in bioassays. Here, we report on the orthologous DAR, *Ag*DOP2, from the malaria mosquito *Anopheles gambiae* Giles that was cloned and pharmacologically characterized in HEK293 cells. Larval bioassays were then conducted to examine the potential of DAR antagonist insecticides against *Anopheles* vectors.

**Findings:**

Previous in vitro cAMP accumulation assays demonstrated Gαs coupling for *Aa*DOP2 and *Cq*DOP2 and dose-dependent inhibition by DAR antagonists. We observed a negligible response of *Ag*DOP2 in the cAMP assay, which prompted an investigation of alternative coupling for mosquito DARs. In an in vitro IP-One Gα_q_ second messenger assay of calcium signaling, dopamine stimulation increased IP1 accumulation in *Aa*DOP2-, *Cq*DOP2- and *Ag*DOP2-expressing cells, and DAR antagonists inhibited IP1 signaling in a dose-dependent manner. In larval bioassays, DAR antagonists caused considerable mortality of *An. gambiae* larvae within 24 h post-exposure.

**Conclusions:**

In vitro data reveal pleiotropic coupling of *Aa*DOP2 and *Cq*DOP2 to Gα_q_ and Gα_s_. In contrast, *Ag*DOP2 appeared to selectively couple to Gαq signaling. In vitro antagonist studies revealed general conservation in pharmacology between mosquito DARs. In vivo data suggest potential for DAR antagonist insecticides against *An. gambiae*. Sequence conservation among the DOP2 receptors from 15 *Anopheles* species indicates utility of antagonists to control residual malaria transmission. *Ag*DOP2 Gα_q_-dependent signaling could be exploited for *An. gambiae* control via pathway specific antagonists.

**Electronic supplementary material:**

The online version of this article (doi:10.1186/s13071-016-1477-6) contains supplementary material, which is available to authorized users.

## Background

Control of malaria transmitted by species of *Anopheles* mosquitoes is largely achieved via long lasting insecticide treated nets and indoor residual sprays. New insecticidal chemistries are needed to protect against mosquitoes that are resistant to existing insecticides. Furthermore, to achieve malaria eradication or elimination, new insecticides are required to disrupt outdoor “residual” transmission by exophilic, day biting mosquitoes [1]. Recently, the Innovative Vector Control Consortium (IVCC; http://www.ivcc.com) issued a call for three new insecticides with novel modes of action by 2023 to control malaria mosquitoes [2]. New products must be mosquito-selective and effective against the many species of *Anopheles* that transmit malaria (see [3]).

Small molecule antagonists of mosquito D_1_-like dopamine receptors (DARs) show promise as a new class of insecticides against the mosquito vectors *Aedes aegypti* and *Culex quinquefasciatus* [4–7]. Several antagonists are potent inhibitors of the *Ae. aegypti Aa*DOP2 and *C. quinquefasciatus Cq*DOP2 DARs in vitro. These chemistries are >100-fold more selective for the mosquito DARs versus the human receptor, hD1, and are highly toxic to mosquito larvae. Further, studies have shown that invertebrate DOP2 receptors are both phylogenetically and pharmacologically distinct from mammalian D_1_-like receptors [8], a significant rationale for targeting of these receptors for insecticides.

Here, building on our previous work for *Aa*DOP2 and *Cq*DOP2, we extend DAR analyses to the *Anophele*s system. The orthologous DAR *Ag*DOP2 was identified from the genome of *Anopheles gambiae*, the mosquito vector of malaria in sub-Saharan Africa, cloned, and pharmacologically characterized. *Ag*DOP2 was expected to exhibit D_1_-like pharmacology based on its relation to other invertebrate dopamine receptors. We present molecular and pharmacological characterization of *Ag*DOP2, as well as larval bioassays that support the potential for developing DAR antagonists to control mosquito vectors of malaria and other devastating human and animal pathogens.

## Findings

### Discovery and molecular characterization of DOP2 DARs from *Anopheles* species

The *Ag*DOP2 gene [GenBank: KU948225] was identified from the *Anopheles gambiae* genome assembly available at VectorBase (https://www.vectorbase.org/) and manual annotation was performed as described by [4]. The conceptual *Ag*DOP2 protein sequence was aligned with *Aa*DOP2 and *Cq*DOP2 using ClustalW [9] (Additional file 1: Figure S1 and Table S1). Residues required for receptor activity and associated with the transmembrane (TM) domains were generally conserved, with greatest divergence observed in the N-terminal region and the intracellular loop 3 (IL3). Of note, the IL3, a region typically associated with coupling to G proteins, is 21 residues longer in *An. gambiae* as compared to *Cx. quinquefasciatus* and *Ae. aegypti*. Gene expression of *Ag*DOP2 in *An. gambiae* developmental stages and sexes was confirmed by RT-PCR, suggesting this receptor, like *Aa*DOP2 and *Cq*DOP2, is constitutively expressed throughout the mosquito life-cycle, and is likely associated with essential neurological processes as in other invertebrates [10]. DOP2 sequences from an additional 14 *Anopheles* species [11] were identified by tBLASTn searches against the GenBank Whole Genome Shotgun Contigs (WGS) database and manual annotation. Alignments revealed between 78.0 and 99.6 % identity of these sequences to *Ag*DOP2 (Additional file 1: Figure S2).

### In vitro Pharmacology of *Ag*DOP2

For functional characterization, *Ag*DOP2 was synthesized by Genscript (Piscataway, NJ, USA), cloned into the expression vector pcDNA3.1+ (Invitrogen, Carlsbad, CA) and a stable cell line expressing the receptor in Human Embryonic Kidney (HEK)-293 cells was generated as previously described [4, 6] by plating cells in a 10 cm dish and transfecting with 15 μl Lipofectamine2000 and 3 μg of plasmid. The pharmacology of *Ag*DOP2 was evaluated in comparison to that of *Aa*DOP2, *Cq*DOP2 and hD1. On the basis of its relationship to other invertebrate dopamine receptors [6] (Additional file 1: Figure S1), *Ag*DOP2 was predicted to couple Gα_s_, a guanine nucleotide binding protein that stimulates adenylyl cyclase activity following receptor activation. However, as the receptor showed no significant response to dopamine in cAMP accumulation assays (See Additional file 1: Figure S3), alternative coupling was investigated using the Cisbio IP-One HTRF accumulation assay (Cisbio, Bedford, MA, USA) that measures receptor activation of Gαq and subsequent stimulation of phospholipase C leading to accumulation of downstream inositol monophosphate (IP1). Assays and analyses were performed as in previous studies for cAMP [5, 6] with the exception that cryopreserved cells were plated in 1X Stimulation Buffer (10 mM HEPES; 1 mM CaCl_2_, 0.5 mM MgCl_2_, 5.5 mM D-Glucose, 4.2 mM KCl, 146 mM NaCl, 50 mM LiCl) and incubated at 37 °C, 5 % CO_2_, and 90 % humidity for 2 h. Drugs were diluted to appropriate concentration in 1X Stimulation Buffer containing 0.02 % ascorbic acid, and added to cells to then incubate for 1 h at 37 °C. Ligand stimulation of cells was arrested by addition of 3 μL/well d2 labelled IP1 and 3 μL/well Cryptate labelled anti-IP1 (diluted 1:5 in lysis buffer). Following incubation for 1 h at room temperature, plates were read on the Synergy 4 (BioTek Instruments, Winooski, VT, USA).

**Fig. 1:**
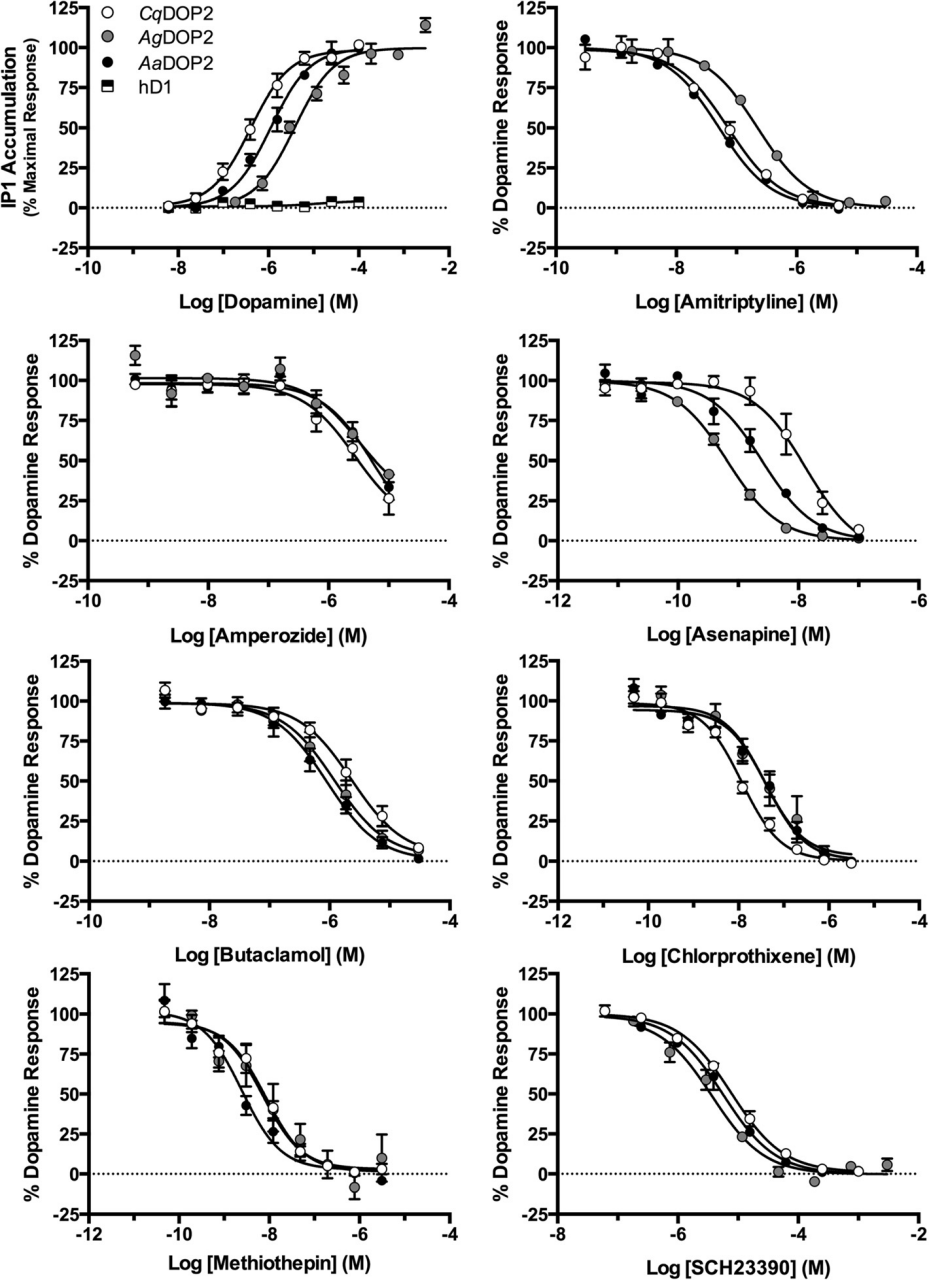
In vitro pharmacological characterization of *Ag*DOP2 using IP1 accumulation assay and comparison to *Aa*DOP2, *Cq*DOP2 and hD1. Cryopreserved cells were plated in 384 well plates (20,000 cells/well), and receptor responses were analyzed for dopamine (upper left panel), or an EC_90_ concentration of dopamine in the presence of the indicated antagonists measured as IP1 accumulation. Data were analyzed using GraphPad prism v.6 software

Increases in intracellular IP1 for each receptor were first measured as concentration response stimulation to dopamine (Fig. 1; Table 1). EC_50_ values revealed that *Aa*DOP2 (1.3 μM ± 0.4) and *Cq*DOP2 (0.7 μM ± 0.2) responded robustly to dopamine stimulation while *Ag*DOP2 (4.7 μM ± 0.4) proved 3 and 7 fold less sensitive. These data suggest mosquito receptors can couple via Gα_q_ in an HEK293 background and reveal a lack of Gα_s_-coupling for *Ag*DOP2 in vitro. As expected, no increase in IP1 accumulation was observed when cells expressing hD1 were treated with dopamine, demonstrating that Gα_q_ coupling does not reflect a general phenomenon for DARs expressed in the in vitro system employed here. Pleiotropic coupling to Gα_s_ and Gα_q_ has been reported for a D_1_-like DAR from the tick, *Ixodes scapularis* and the honey bee, *Apis mellifera* [12, 13]. Similar studies with the *Drosophila melanogaster* D_1_-like receptor, DopR99B, also implicate multiple second messenger systems [14] and the involvement of Gα_q_, Gα_i/o_- and Gβγ-coupling [15]. While hD1 couples only via Gα_s_, other human G protein coupled receptors (GPCRs) can signal via multiple G proteins [16, 17]. Further studies are required to confirm pleiotropic coupling of mosquito DARs in an insect cell background and in vivo, as well as to explore potential divergence between the signaling mechanisms of invertebrate and mammalian DARs. Apparent dependence of *Ag*DOP2 on Gα_q_-coupling in vitro was an unexpected finding that may enable the identification of residues determining G protein interactions and development of products that selectively disrupt Gαq-mediated signaling of DOP2 in mosquitoes.

**Table 1:** IC_50_ values (μM ± SEM) for inhibition of dopamine-stimulated IP1 response in HEK-293 cell lines by DAR antagonists

Compound	*Ag*DOP2	*Aa*DOP2	*Cq*DOP2	Fold selectivity to *Ag*DOP2	
				*Aa*DOP2	*Cq*DOP2
Amitriptyline	0.23 ± 0.02	0.05 ± 0.003	0.08 ± 0.007	4.4	3.0
Amperozide	18.8 ± 11	5.1 ± 1	2.6 ± 0.6	3.7	7.2
Asenapine	0.0007 ± 0.00008	0.003 ± 0.0005	0.02 ± 0.004	0.3	0.05
Butaclamol	1.2 ± 0.4	1.0 ± 0.2	2.8 ± 0.9	1.2	0.4
Chlorprothixene	0.4 ± 0.007	0.04 ± 0.01	0.01 ± 0.002	0.9	3.2
Methiothepin	0.14 ± 0.008	0.003 ± 0.001	0.01 ± 0.005	4.1	1.1
SCH23390	3.6 ± 0.8	4.7 ± 0.8	8.3 ± 1	0.8	0.4

The mosquito DARs exhibited similar profiles in response to DAR antagonists (Fig. 1; Table 1), suggesting a general conservation in receptor pharmacology. A suitable signal window was produced for these antagonist studies by stimulating the receptor-expressing HEK cells with an EC_90_ concentration of dopamine (10 μM for *Aa*DOP2 and *Cq*DOP2 and 100 μM for *Ag*DOP2). Of the antagonists analyzed, amitriptyline, amperozide, chlorprothixene and methiothepin showed a higher potency at both *Aa*DOP2 (4–35 fold) and *Cq*DOP2 (3–40 fold), than at *Ag*DOP2. Asenapine followed by SCH23390, a standard pharmacological probe used in previous investigations [4–6], proved the most potent for *Ag*DOP2. Alternatively, butaclamol demonstrated slightly higher potency for *Aa*DOP2 and *Ag*DOP2, than *Cq*DOP2.

### Toxicity of DOP2 antagonists to *Anopheles gambiae* larvae

**Fig. 2:**
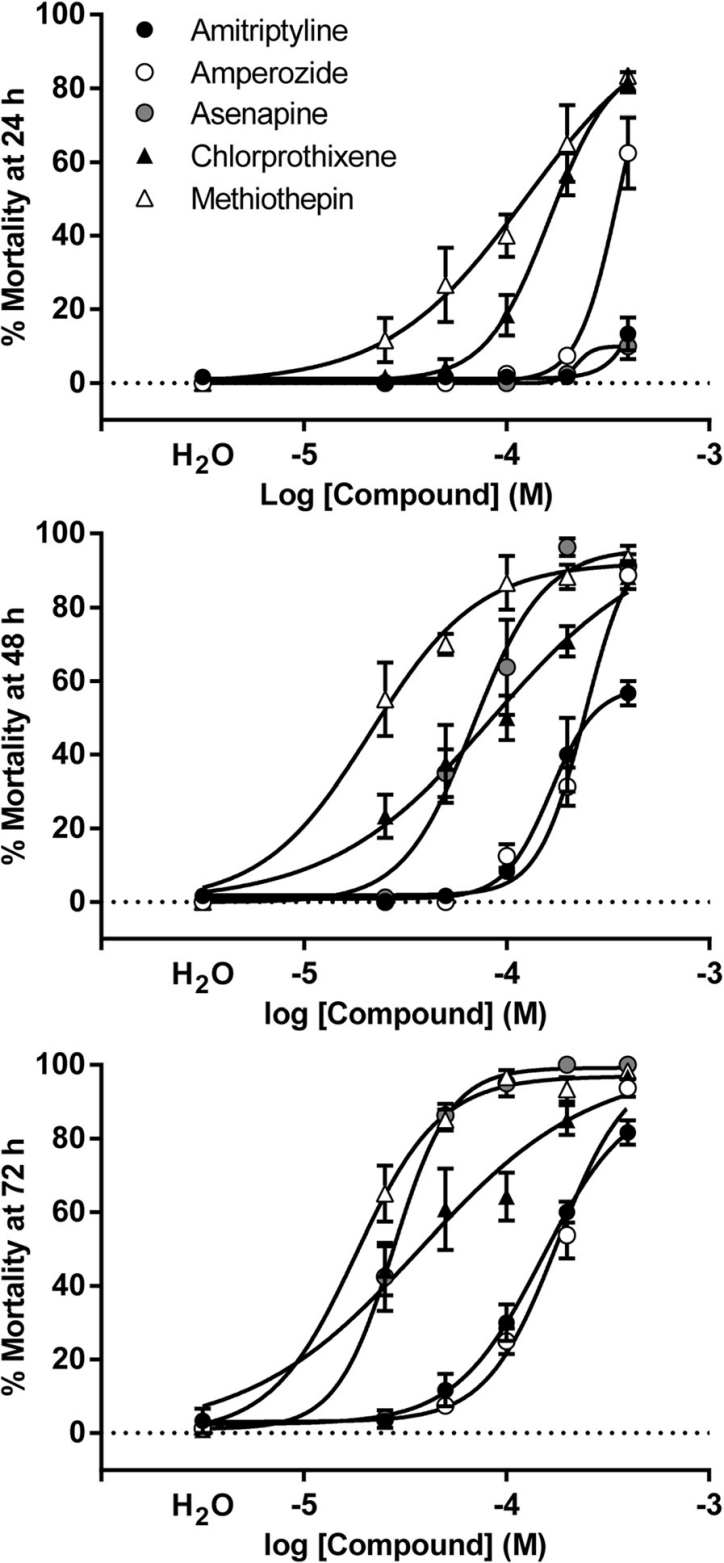
Concentration response curves for *An. gambiae* showing percent larval mortality at 24, 48 and 72 h post exposure to DOP2 antagonists; Each data point represents mean ± SEM (*n* ≥ 3 independent experiments)

As in previous work with *Aedes* and *Culex* [5], we observed a correlation between in vitro and in vivo results in the *Anopheles* system. The in vivo activity of select antagonists was tested in L3 *An. gambiae* larvae, using concentration response assays conducted at 26 °C as described by [6] (note: SCH23390 was not included as this chemistry had no toxicity to *Aedes* and *Culex* larvae). Larvae of the KISUMU1 strain obtained through the MR4 (MRA catalog number MRA-762, KISUMU1 F34 strain, established by Dr. G. Davidson, donated by Vincent Corbel) were reared on a 12 h day/night cycle at 75 % RH at 28 °C in 25 × 40 cm plastic pans (400 larvae per pan) on a diet of ground flake fish food. Antagonists were selected based on demonstrated toxicity to L3 larvae of *Ae. aegypti* and *C. quinquefasciatus* [6]. DAR antagonists caused mortality of *An. gambiae* larvae 24 h post exposure (Fig. 2; Table 2). Methiothepin, asenapine and chlorprothixene were among the most toxic compounds at 72 h as compared to amitriptyline (LC_50_ = 151 μM), the chemistry employed as positive control in *Ae. aegypti* and *Cu. quinquefasciatus* bioassays [4, 5]. Amitriptyline was also identified by [18] as toxic to *An. gambiae* larvae and adults. Methiothepin and chlorprothixene were the most rapidly toxic to *An. gambiae,* presumably due to physico-chemical properties that affect absorption as discussed by [6]. Asenapine caused negligible toxicity at 24 h but toxicity was observed by 48 h. Chlorprothixene caused mortality (LC_50_ = 163 μM) initially, although most survivors remained viable for several days. The high sequence conservation between the DOP2 receptors of 14 *Anopheles* spp. from sub-Saharan Africa, south-east Asia and Latin America suggests the DAR antagonists identified may be broadly active at the DOP2 receptors of malaria vector species, including those that contribute significantly to residual malaria transmission. Genome assemblies for multiple *Anopheles* species [11] and populations [19] offer the opportunity to expand comparative molecular and pharmacological studies of DAR targets across the subfamily Anophelinae.

**Table 2:** Toxicity of DOP2 antagonists to larvae of *An. gambiae* showing lethal concentration (LC_50_) values (μM ± SEM) (*n* ≥ 3)

	24 h	48 h	72 h
Amitriptyline	*N.A.* ^a^	163 ± 22	151 ± 28
Amperozide	*N.A.* ^a^	245 ± 68	182 ± 24
Asenapine	*N.A.* ^a^	69 ± 15	28 ± 5
Chlorprothixene	163 ± 17	128 ± 84	112 ± 72
Methiothepin	137 ± 110	51 ± 30	*N.A.* ^b^

Mortality too low (^a^) or high (^b^) to accurately calculate LC_50_

## Conclusions

We present evidence of pleiotropic coupling via Gα_s_ and Gα_q_ among the mosquito DARs, *Aa*DOP2 and *Cq*DOP2. In contrast, *Ag*DOP2 appeared to selectively couple to Gα_q_ signaling in vitro. The heterologous expression studies also revealed general conservation in pharmacology between mosquito DARs including their relatively similar responses to DAR antagonists. Asenapine was the most potent and selective *Ag*DOP2 antagonist in vitro and caused mortality of *An. gambiae* larvae. This and other antagonists offer “probes” for further pharmacological investigations. While physiochemical properties such as low lipophilicity and the presence of a charged amine group at physiological pH may limit the application of these chemistries as insecticidal leads, they never the less offer an important starting point for discovery of derivatives effective against *Anopheles* mosquitoes. Sequence conservation among the DOP2 DARs of 14 *Anopheles* species suggests potential to develop products to control residual transmission of malaria by multiple vectors. The discovery of an additional signaling pathway for mosquito DARs may offer opportunities to disrupt dopaminergic physiology of these vectors with new chemistries likely active through complex mechanisms.

## Additional file

Additional file 1: Genomic and pharmacologic assessment of mosquito dopamine receptors. (DOCX 1422 kb)

## Abbreviations

*Aa*DOP2: *Aedes aegypti* dopamine receptor 2
*Ag*DOP2: *Anopheles gambiae* dopamine receptor 2
cAMP: cyclic adenosine monophosphate
*Cq*DOP2: *Culex quinquefasciatus* dopamine receptor 2
DAR: dopamine receptor
EC: effective concentration
EL: extracellular loop
Gα_s_: G protein subunit that stimulates adenylyl cyclase
Gα_q_: G protein subunit that stimulates phospholipase C
GPCR: G protein-coupled receptor
HEK: human embryonic kidney cells
hD1: human D_1_-like dopamine receptor
HTRF: homogenous time resolved fluorescence
IC_50_: inhibitory concentration
IL: intracellular loop
IP1: inositol monophosphate
IRS: indoor residual spray
LC_50_: lethal concentration
LLIN: long lasting insecticide treated net
MoA: mode of action
PLC: phospholipase C
TM: transmembrane domain
