# Supplementary material for: Comparative pharmacological characterization of D1-like dopamine receptors from Anopheles gambiae, Aedes aegypti and Culex quinquefasciatus suggests pleiotropic signaling in mosquito vector lineages
Source: Parasit Vectors. 2016 Apr 6;9:192. doi: 10.1186/s13071-016-1477-6 (PMC4822259; doi:10.1186/s13071-016-1477-6)
Supplement: Additional file 1: — Genomic and pharmacologic assessment of mosquito dopamine receptors. (DOCX 1422 kb) [file 13071_2016_1477_MOESM1_ESM.docx]

**Additional File 1**

**Table S1. Comparison of protein features for DOP2 of *Anopheles gambiae*, *Culex quinquefasciatus*, and *Aedes aegypti.*** The number of amino acids composing the N- and C-termini and the intracellular and extracellular loops are relative to the transmembrane domain (TM) sequences in Figure 1 (Additional file 1).

| **Protein features** | **Amino Acids** | | |
| --- | --- | --- | --- |
|  | ***Ag*DOP2** | ***Cq*DOP2^a^** | ***Aa*DOP2^b^** |
| Total length | 495 | 479 | 476 |
| Length of N-terminus | 54 | 58 | 57 |
| Length of intracellular loops I, II, III | 10, 20, 137 | 10, 20, 117 | 10, 20, 115 |
| Length of extracellular loops I, II, III | 15, 18, 9 | 15, 18, 9 | 15, 18, 9 |
| Length of carboxyl tail | 63 | 63 | 63 |
| 1-4 *N*-linked glycosylation sites (N-terminus) | N4, N20, N25, N43 | N,5, N21, N26, N47 | N3, N19, N24, N46 |
| Conserved cysteines in extracellular loops 1-2 | C129, C208 | C133, C212 | C132, C211 |
| C-terminus palmitoylation sites (C) | C447, C448 | C387, C431, C432, C457 | C384, C428, C429, C454 |
| Protein kinase A/C phosphorylation (Intracellular loops II, III and C-terminus) | S163, S249,T275, S307, T310, T324, S358, S360, T364, T459, S462, S465, S470, S475, T476, S478, S489 | S167, T173, T251, S253, T279, T307, S342, S344, T443, S449, S454, S459, T460, S462, S473 | S166, T172, T250, S252, T278, T305, S339, S341, T440, S446, S451, S456, T457, S459, S470 |
| Conserved aspartates in TMII, TMIII | D101, D136 | D105, D140 | D104, D139 |
| Conserved “DRY” motif | D153, R154, Y155 | D157, R158, Y159 | D156, R157, Y158 |
| Conserved serines in TMV | S220, S221, S224 | S224, S225, S228 | S223, S224, S227 |
| Conserved aromatic residue in TMV | F225 | F229 | F228 |
| Conserved aromatic residues in TMVI | W387, F390, F391 | W371, F374, F375 | W368, F371, F372 |

^a^[6]; ^b^[4]

**
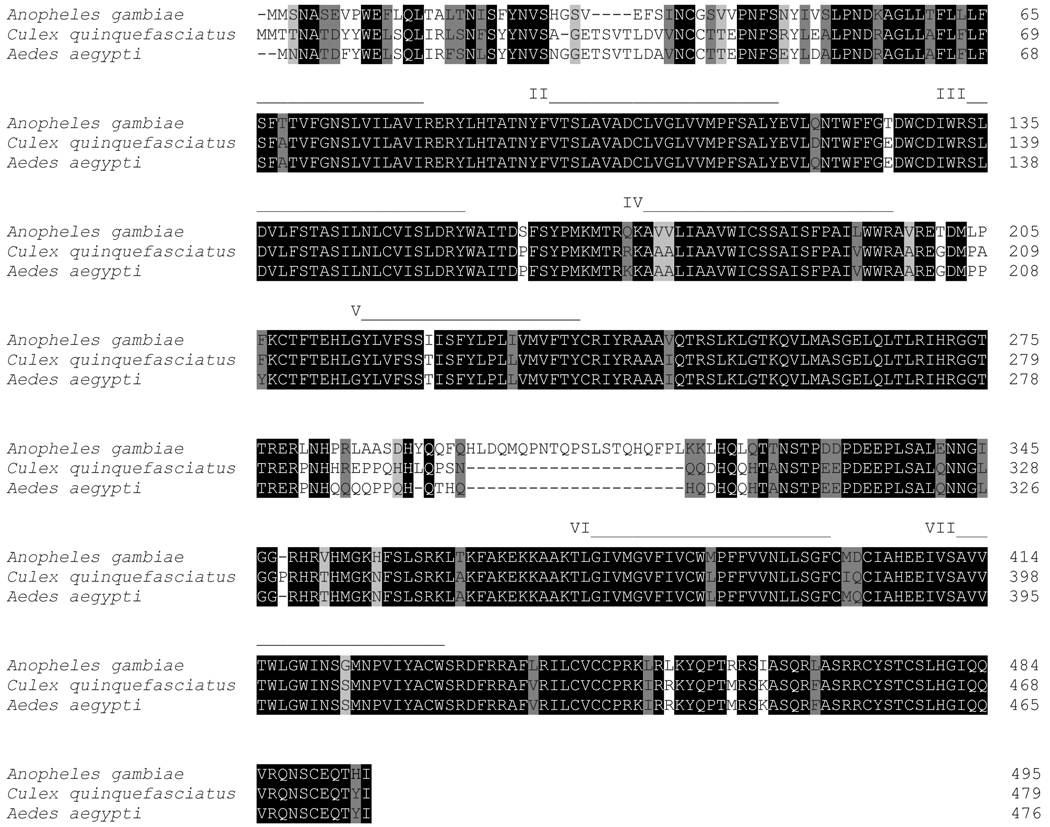
**

**Figure S1. Alignment of *Ag*DOP2 with *Aa*DOP2 and *Cq*DOP2.** Note the third intracellular loop (IL3) of *Ag*DOP2 is 21 residues longer than that of *Aa*DOP2 and *Cq*DOP2. Highlighted areas indicate identical and conserved residues as designated by ClustalW [8]. Black shading = identical residues; dark gray shading = strongly similar residues; light gray shaing = weakly similar residues (for amino acid similarity groups, see: <http://www.clustal.org/download/clustalx_help.html>). Putative transmembrane (TM) domains I-VII are indicated as a line above the alignment.


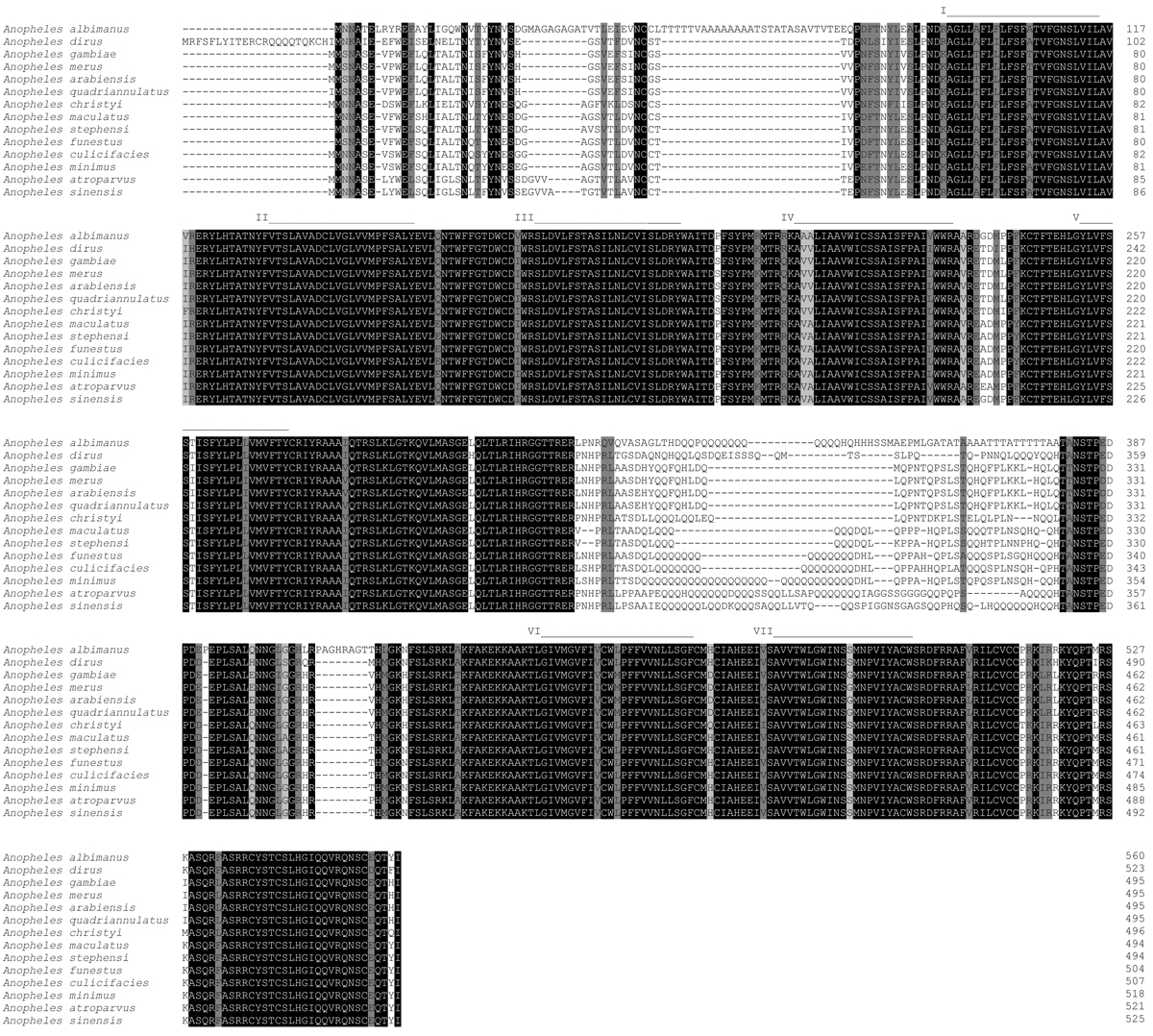


**Figure S2. Alignment of conceptual DOP2 DAR sequences from 14 *Anopheles* species**. Highlighted areas indicate identical and conserved residues as designated by ClustalW [8]. Black shading = identical residues; dark gray shading = strongly similar residues; light gray shaing = weakly similar residues (for amino acid similarity groups, see: http://www.clustal.org/download/clustalx_help.html). Conceptual DOP2 sequences were predicted following tBLASTn search against the Genbank Whole Genome Shotgun Contigs (wgs) database, followed by manual curation to obtain the gene model. The WGS assemblies for each species are as follows: *Anopheles albimanus*, [GenBank: APCK01001250]; *An. arabiensis*, [GenBank: APCN01001073]; *An. atroparvus*, [GenBank: AXCP01006173; AXCP01006172]; *An. christyi,* [GenBank: APCM01008275; APCM01012391; APCM01010113]; *An. culicifacies*, [GenBank: AXCM01006973; AXCM01006974]; *An. dirus*, [GenBank: APCL01007738; APCL01007754; APCL01007860]; *An. funestus,* [GenBank: APCI01005391; APCI01005390]; *An. gambiae* M form, [GenBank: ABKP02003382; ABKP02020596]; *An. koliensis,* [GenBank: JXXB01004691; JXXB01037766; JXXB01012835]*; An. maculatus*, [GenBank: AXCL01012651; AXCL01039937; AXCL01008884]; *An. merus*, [GenBank: AXCQ02011090; AXCQ02012580]; *An. minimus*, [GenBank: APHL01001783]; *An. sinensis*, [GenBank: AXCK02003865; ATLV01017198]; *An. stephensi*, [GenBank: ALPR02007977; APCG01006541; APCG01006537]; *An. quadriannulatus*, [GenBank: APCH01004885].

**Figure S3.** ***In vitro* pharmacological analysis of cAMP accumulation in response to dopamine stimulation of *Aa*DOP2, *Cq*DOP2, and *Ag*DOP2.** Cryopreserved cells were plated in 384 well plates, stimulated with dopamine (100 µM), and cAMP accumulation was measured using Cisbio HTRF reagents. Further assay details, are described in [5]. Average fold-stimulation of dopamine over vehicle was 21 fold (*Aa*DOP2), 65 fold (*Cq*DOP2), and 1.6 fold (*Ag*DOP2). Data were analyzed using GraphPad prism v.6 software (data represent the average ± S.E.M. of 6-7 independent experiments).

**Reagents for *In vitro* Studies**

Reagents for *in vitro* and *in vivo* studies were obtained from the following suppliers: Amitriptyline HCl, Ascorbic Acid, Asenapine Maleate, (+) Butaclamol HCl, Calcium Chloride Dihydrate, Dopamine HCl, Lithium Chloride, Magnesium Chloride Hexahydrate, Potassium Chloride, R(+)-SCH-23390 HCl, Sodium Chloride were all purchased from Sigma Aldrich (St. Louis, MO). D-Glucose was purchased from Mallinckrodt Baker Inc. (Paris, KY). 1M HEPES was purchased from Thermo Scientific (Waltham, MA). Bovine Calf Serum, Dulbecco’s Modified Eagles Media (DMEM), Fetal Clone I, G418, Opti-MEM, Phosphate Buffered Saline (PBS), Penicillin-Streptomycin, 5% Trypsin, were purchased from Life Technologies (Grand Island, NY). The HTRF IP-One kit was purchased from Cisbio Bioassays (Bedford, MA). 384 well small volume assay plates were purchased from Greiner Bio-One (Monroe, NC).

**Identification of DOP2 Sequences from *Anopheles* spp.**

The alignment shown in Figure 2 was generated using sequences for *Anopheles* spp. vectors of malaria from sub-Saharan Africa, South Asia, and SE Asia/Latin America that exhibit endophagic/exophagic and zoophagic/anthropophagic behaviors as follows: *An. arabiensis* (sub-Saharan Africa, endophagic), *An. stephensi* (Sth Asia; strongly exophagic and zoophagic), *An. culicifacies* (Sth Asia, strongly zoophagic and exophagic), *An. funestus* (Sub-Saharan Africa, anthropophagic and endophagic) and *An. albimanus* (SE Asia/Latin American; highly endophagic and moderately-strongly zoophagic as per [3,10].

**Conceptual Protein Sequence of DOP2 DARs Identified From 15 *Anopheles* Species**

>AalbDOP2

MNNATELRYREFAYLIGQWNVTYYNVSDGMAGAGAGATVTLEIEVNCCLTTTTTVAAAAAAAATSTATASAVTVTEEQPDFTNYLEALPNDRAGLLAFLFLFSFATVFGNSLVILAVVRERYLHTATNYFVTSLAVADCLVGLVVMPFSALYEVLQNTWFFGTDWCDVWRSLDVLFSTASILNLCVISLDRYWAITDPFSYPMRMTRRKAAALIAAVWICSSAISFPAIVWWRAARDGDMPPFKCTFTEHLGYLVFSSTISFYLPLLVMVFTYCRIYRAAAIQTRSLKLGTKQVLMASGELQLTLRIHRGGTTRERLPNRQVQVASAGLTHDQQPQQQQQQQQQQQHQHHHSSMAEPMLGATATAAAATTTATTTTTAATANSTPEDPDEPEPLSALQNNGLGGHLRPAGHRAGTTHLGKNFSLSRKLAKFAKEKKAAKTLGIVMGVFIVCWLPFFVVNLLSGFCMHCIAHEEIVSAVVTWLGWINSSMNPVIYACWSRDFRRAFVRILCVCCPRKIRRKYQPTMRSKASQRFASRRCYSTCSLHGIQQVRQNSCEQTYI

>AaraDOP2

MMSNASEVPWEFLQLTALTNISFYNVSHGSVEFSINCGSVVPNFSNYIVSLPNDKAGLLTFLLLFSFTTVFGNSLVILAVIRERYLHTATNYFVTSLAVADCLVGLVVMPFSALYEVLQNTWFFGTDWCDIWRSLDVLFSTASILNLCVISLDRYWAITDSFSYPMKMTRQKAVVLIAAVWICSSAISFPAILWWRAVRETDMLPFKCTFTEHLGYLVFSSIISFYLPLIVMVFTYCRIYRAAAVQTRSLKLGTKQVLMASGELQLTLRIHRGGTTRERLNHPRLAASEHYQQFQHLDQLQPNTQPSLSTQHQFPLKKLHQLQTTNSTPDDPDEEPLSALENNGIGGRHRVHMGKHFSLSRKLTKFAKEKKAAKTLGIVMGVFIVCWMPFFVVNLLSGFCMDCIAHEEIVSAVVTWLGWINSGMNPVIYACWSRDFRRAFLRILCVCCPRKLRLKYQPTRRSIASQRLASRRCYSTCSLHGIQQVRQNSCEQTHI

>AatrDOP2

MMNNASELYWELSQLIGLSNLTFYNVSSDGVVAGTVTLAVNCCTTEPNFSNYLESLPNDRAGLLAFLFLFSFATVFGNSLVILAVIRERYLHTATNYFVTSLAVADCLVGLVVMPFSALYEVLQNTWFFGTDWCDIWRSLDVLFSTASILNLCVISLDRYWAITDPFSYPMKMTRRKAVALIAAVWICSSAISFPAIVWWRAAREEAMPPFKCTFTEHLGYLVFSSTISFYLPLLVMVFTYCRIYRAAAIQTRSLKLGTKQVLMASGELQLTLRIHRGGTTRERPNHPRLLPPAAPEQQQHQQQQQQDQQQSSQQLLSAPQQQQQQQIAGGSSGGGGQQPQPSAQQQQHTANSTPEDPDDEPLSALQNNGLGGRHRPHMGKNFSLSRKLAKFAKEKKAAKTLGIVMGVFIVCWLPFFVVNLLSGFCMHCIAHEEIVSAVVTWLGWINSSMNPVIYACWSRDFRRAFVRILCVCCPRKIRRKYQPTMRSKASQRFASRRCYSTCSLHGIQQVRQNSCEQTYI

>AchrDOP2

MMNNASEDSWEFLKLIELTNVSYYNESQGAGFVKLDSNCCSVVPNFSNFIISLPNDKAGLLAFLFLFSFATVFGNSLVILAVFRERYLHTATNYFVTSLAVADCLVGLVVMPFSALYEVLQNTWFFGTDWCDIWRSLDVLFSTASILNLCVISLDRYWAITDSFSYPMKMTRRKAVVLIAAVWICSSAISFPAILWWRAVRETDMIPFKCTFTEHLGYLVFSSIISFYLPLIVMVFTYCRIYRAAAVQTRSLKLGTKQVLMASGELQLTLRIHRGGTTRERPNHPRLATSDLLQQQLQQLEQLQPNTDKPLSTELQLPLNNQQLTANSTPEDPDDEPLSALQNNGLGGRHRVHMGKHFSLSRKLTKFAKEKKAAKTLGIVMGVFIICWLPFFVVNLLSGFCMQCIAHEEIISAVVTWLGWINSSMNPVIYACWSRDFRRAFVRILCVCCTKKIRRKYQPTLRSMASQRLASRRCYSTCSLHGIQQVRQNSCEQTQI

>AculDOP2

MMNNASEVSWEFSQLIALTNQSYYNESGGAGSVTLDVNCCTIVPDFTNYLESLPNDKAGLLAFLFLFSFATVFGNSLVILAVIRERYLHTATNYFVTSLAVADCLVGLVVMPFSALYEVLENTWFFGTDWCDIWRSLDVLFSTASILNLCVISLDRYWAITDPFSYPMKMTRRKAVALIAAVWICSSAISFPAILWWRAVREADMPPYKCTFTEHLGYLVFSSTISFYLPLLVMVFTYCRIYRAAAIQTRSLKLGTKQVLMASGELQLTLRIHRGGTTRERLSHPRLTASDQLQQQQQQQQQQQQQQQDHLQPPAHHQPLATQQQSPLNSQHQPHTANSTPEDPDDEPLSALQNNGLGGRHRTHMGKNFSLSRKLAKFAKEKKAAKTLGIVMGVFIVCWLPFFVVNLLSGFCMHCIAHEEIVSAVVTWLGWINSSMNPVIYACWSRDFRRAFVRILCVCCPRKIRRKYQPTMRSKASQRFASRRCYSTCSLHGIQQVRQNSCEQTYI

>AdirDOP2

MRFSFLYITERCRQQQQTQKCHIMNKAIEEFWEISELNELTNYTYYNVSEGSVTFDVNCSTTDPNLSIYIESLPNDRAGLLAFLILFSFATVFGNSLVILAVIHERYLHTATNYFVTSLAVADCLVGLVVMPFSALYEVLQNTWFFGTDWCDIWRSLDVLFSTASILNLCVISLDRYWAITDPFSYPMKMTRRKAVVLIAAVWICSSAISFPAIIWWRAVRETDIPPFKCTFTEHLGYLVFSSTISFYLPLLVMVFTYCRIYRAAAIQTRSLKLGTKQVLMASGEHQLTLRIHRGGTTRERPNHPRLTGSDAQNQHQQLQSDQEISSSQQMTSSLPQTQPNNQLQQQYQQHTANSTPEDPDDEPLSALHNNGLSGRQRMHMGKNFSLSRKLAKFAKEKKAAKTLGIVMGVFIVCWLPFFVVNLLSGFCMHCIAHEEIVSAVVTWLGWINSSMNPVIYACWSRDFRRAFVRILCVCCPRKIKHKYQPTIRSKASQRFASRRCYSTCSLHGIQQVRQNSCDQTFI

>AfunDOP2

MSNASEVFWEFSQLIALTNQTYNESDGAGSVTLDVNCCTIVPDFTNYLESLPNDKAGLLAFLFLFSFATVFGNSLVILAVIRERYLHTATNYFVTSLAVADCLVGLVVMPFSALYEVLENTWFFGTDWCDIWRSLDVLFSTASILNLCVISLDRYWAITDPFSYPMKMTRRKAVALIAAVWICSSAISFPAILWWRAVREADMPPYKCTFTEHLGYLVFSSTISFYLPLLVMVFTYCRIYRAAAIQTRSLKLGTKQVLMASGELQLTLRIHRGGTTRERLNHPRLAASDQLQQQQQQQQQQQQQQDHLQPPAHQPLSAQQQSPLSGQHQQQHTANSTPEDPDDEPLSALQNNGLGGRHRTHMGKNFSLSRKLAKFAKEKKAAKTLGIVMGVFIVCWLPFFVVNLLSGFCMHCIAHEEIVSAVVTWLGWINSSMNPVIYACWSRDFRRAFVRILCVCCPRKIRRKYQPTMRSKASQRFASRRCYSTCSLHGIQQVRQNSCEQTYI

>AgamDOP2

MMSNASEVPWEFLQLTALTNISFYNVSHGSVEFSINCGSVVPNFSNYIVSLPNDKAGLLTFLLLFSFTTVFGNSLVILAVIRERYLHTATNYFVTSLAVADCLVGLVVMPFSALYEVLQNTWFFGTDWCDIWRSLDVLFSTASILNLCVISLDRYWAITDSFSYPMKMTRQKAVVLIAAVWICSSAISFPAILWWRAVRETDMLPFKCTFTEHLGYLVFSSIISFYLPLIVMVFTYCRIYRAAAVQTRSLKLGTKQVLMASGELQLTLRIHRGGTTRERLNHPRLAASDHYQQFQHLDQMQPNTQPSLSTQHQFPLKKLHQLQTTNSTPDDPDEEPLSALENNGIGGRHRVHMGKHFSLSRKLTKFAKEKKAAKTLGIVMGVFIVCWMPFFVVNLLSGFCMDCIAHEEIVSAVVTWLGWINSGMNPVIYACWSRDFRRAFLRILCVCCPRKLRLKYQPTRRSIASQRLASRRCYSTCSLHGIQQVRQNSCEQTHI

>AkolDOP2

MNNVIDEFWDTLDFKRLANYTYYNASEELVTLDVNFSTTDPNFSIYIETLSNDRASLLAFLILFSFATVFGNSLVILAVIRERYLHTATNYFVTSLAVADCLVGLVVMPFSALYEVLQNTWFFGTDWCDIWRSLDVLFSTASILNLCVISLDRYWAITDPFSYPMKMTRRKAVALIAAVWICSSAISFPAIVWWRAVREIDIPAFKCTFTEHLGYLVFSSTISFYFPLLVMVFTYYRIYRAAAIQTRSLKLGTKQVLMASGELQLTLRIHRGGTTRERSTHPRMAGSDVQNQNQQLQSDQQISPSQQLVSSIPQTKPNIQMQQQYQQHTANSTPEDQEDEPLSALHNNGISGRNRMHMGKNFSLSRKIAKFAKEKKAAKTLGIVMGVFIVCWLPFFVINLLSGFCVHCIAHEEIVSAVVTWLGWINSSMNPVIYACWSRDFRRAFVRILCVCCPKKIKRKYQPTMRSKASQRFASRRCYSTCSLHGIQQVRQNSCEQTLI

>AmacDOP2

MNNASEVFWEFSHLIALTNLTYYNESDGAGSVTLDVNCCSIVPDFTNYLESLPNDKAGLLAFLFLFSFATVFGNSLVILAVIRERYLHTATNYFVTSLAVADCLVGLVVMPFSALYEVLENTWFFGTDWCDIWRSLDVLFSTASILNLCVISLDRYWAITDPFSYPMKMTRRKAVALIAAVWICSSAISFPAILWWRAVREADMPPYKCTFTEHLGYLVFSSTISFYLPLLVMVFTYCRIYRAAAIQTRSLKLGTKQVLMASGELQLTLRIHRGGTTRERVPRLTAADQLQQQQQQDQLQPPPHQPLSSQQQTPLNSQHQQHTANSTPEDPDDEPLSALQNNGLAGRHRTHMGKNFSLSRKLAKFAKEKKAAKTLGIVMGVFIVCWLPFFVVNLLSGFCMHCIAHEEIVSAVVTWLGWINSSMNPVIYACWSRDFRRAFVRILCVCCPRKIRRKYQPTMRSKASQRFASRRCYSTCSLHGIQQVRQNSCEQTY

>AmerDOP2

MMSNASEVPWEFLQLTALTNISFYNVSHGSVEFSINCGSVVPNFSNYIVSLPNDKAGLLTFLLLFSFTTVFGNSLVILAVIRERYLHTATNYFVTSLAVADCLVGLVVMPFSALYEVLQNTWFFGTDWCDIWRSLDVLFSTASILNLCVISLDRYWAITDSFSYPMKMTRQKAVVLIAAVWICSSAISFPAILWWRAVRETDMLPFKCTFTEHLGYLVFSSIISFYLPLIVMVFTYCRIYRAAAVQTRSLKLGTKQVLMASGELQLTLRIHRGGTTRERLNHPRLAASDHYQQFQHLDQLQPNTQPSLSTQHQFPLKKLHQLQTTNSTPDDPDEEPLSALENNGIGGRHRVHMGKHFSLSRKLTKFAKEKKAAKTLGIVMGVFIICWMPFFVVNLLSGFCMDCIAHEEIVSAVVTWLGWINSGMNPVIYACWSRDFRRAFLRILCVCCPRKLRLKYQPTRRSIASQRLASRRCYSTCSLHGIQQVRQNSCEQTHI

>AminDOP2

MNNASEVSWEFSQLIALTNQTYYNESEGAGSVTLDVNCCTIVPEFTNYLESLPNDKAGLLAFLFLFSFATVFGNSLVILAVIRERYLHTATNYFVTSLAVADCLVGLVVMPFSALYEVLENTWFFGTDWCDIWRSLDVLFSTASILNLCVISLDRYWAITDPFSYPMKMTRRKAVALIAAVWICSSAISFPAILWWRAVREADMPPFKCTFTEHLGYLVFSSTISFYLPLLVMVFTYCRIYRAAAIQTRSLKLGTKQVLMASGELQLTLRIHRGGTTRERLSHPRLTTSDQQQQQQQQQQQQQQQQQQQQQQQQQQQQQQDHLQPPAHQPLSTQPQSPLNSQHQQHTANSTPEDPDDEPLSALQNNGLGGRHRTHMGKNFSLSRKLAKFAKEKKAAKTLGIVMGVFIVCWLPFFVVNLLSGFCMHCIAHEEIVSAVVTWLGWINSSMNPVIYACWSRDFRRAFVRILCVCCPRKIRRKYQPTMRSKASQRFASRRCYSTCSLHGIQQVRQNSCEQTYI

>AquaDOP2

IMSNASEVPWEFLQLTALTNISFYNVSHGSVEFSINCGSVVPNFSNYIVSLPNDKAGLLTFLLLFSFTTVFGNSLVILAVIRERYLHTATNYFVTSLAVADCLVGLVVMPFSALYEVLQNTWFFGTDWCDIWRSLDVLFSTASILNLCVISLDRYWAITDSFSYPMKMTRQKAVVLIAAVWICSSAISFPAILWWRAVRETDMLPFKCTFTEHLGYLVFSSIISFYLPLIVMVFTYCRIYRAAAVQTRSLKLGTKQVLMASGELQLTLRIHRGGTTRERLNHPRLAASEHYQQFQHLDQLQPNTQPSLSTQHQFPLKKLHQLQTTNSTPDDPDEEPLSALENNGIGGRHRVHMGKHFSLSRKLTKFAKEKKAAKTLGIVMGVFIVCWMPFFVVNLLSGFCMDCIAHEEIVSAVVTWLGWINSGMNPVIYACWSRDFRRAFLRILCVCCPRKLRLKYQPTRRSIASQRLASRRCYSTCSLHGIQQVRQNSCEQTHI

>AsinDOP2

MMNNASELYWELSQLIGLSNLTFYNVSSEGVVATGTVTLAVNCCTTEPNFSNYLESLPNDRAGLLAFLFLFSFATVFGNSLVILAVIRERYLHTATNYFVTSLAVADCLVGLVVMPFSALYEVLQNTWFFGTDWCDIWRSLDVLFSTASILNLCVISLDRYWAITDPFSYPMKMTRRKAVALIAAVWICSSAISFPAIVWWRAAREGDMPPFKCTFTEHLGYLVFSSTISFYLPLLVMVFTYCRIYRAAAIQTRSLKLGTKQVLMASGELQLTLRIHRGGTTRERPNHPRLLPSAAIEQQQQQQLQQDKQQQSAQQLLVTQQQSPIGGNSGAGSQQPHQSQLHQQQQQQHQQHTANSTPEDPDDEPLSALQNNGLGGRHRTHMGKNFSLSRKLAKFAKEKKAAKTLGIVMGVFIVCWLPFFVVNLLSGFCMHCIAHEEIVSAVVTWLGWINSSMNPVIYACWSRDFRRAFVRILCVCCPRKIRRKYQPTMRSKASQRFASRRCYSTCSLHGIQQVRQNSCEQTYI

>AsteDOP2

MNNASEVFWELSQLTALTNLTYYNESDGAVSVTLDVNCCTIVPDFTNYLESLSNDKAGLLAFLFLFSFATVFGNSLVILAVIRERYLHTATNYFVTSLAVADCLVGLVVMPFSALYEVLENTWFFGTDWCDIWRSLDVLFSTASILNLCVISLDRYWAITDPFSYPMKMTRRKAVALIAAVWICSSAISFPAILWWRAVREADMPPYKCTFTEHLGYLVFSSTISFYLPLLVMVFTYCRIYRAAAIQTRSLKLGTKQVLMASGELQLTLRIHRGGTTRERVPRLTASDQLQQQQQQDQLKPPAHQPLSSQQHTPLNNPHQQHTANSTPEDPDDEPLSALQNNGLAGRHRTHMGKNFSLSRKLAKFAKEKKAAKTLGIVMGVFIVCWLPFFVVNLLSGFCMHCIAHEEIVSAVVTWLGWINSSMNPVIYACWSRDFRRAFVRILCVCCPRKIRRKYQPTMRSKASQRFASRRCYSTCSLHGIQQVRQNSCEQTYI

Abbreviations: *An. albimanus,* Aalb; *An. arabiensis,* Aara; *An. atroparvus*, Aatr; *An. christyi,* Achr; *An. stephensi*, Aste; *An. culicifacies,* Acul; *An.* dirus, Adir; An*. funestus,* Afun; *An. gambiae,* Agam; *An. koliensis,* Akol*; An. maculatus*, Amac; *An. merus*, Amer; *An. minimus*, Amin; *An. quadriannulatus*, Aqua; *An. sinensis*, Asin; *An. stephensi*, Aste.
